# Effect of Hormonization Treatment on Yield Quantity and Quality, Contents of Biologically Active Compounds, and Antioxidant Activity in ‘Einset Seedless’ Grapevine Fruits and Raisins

**DOI:** 10.3390/molecules26206206

**Published:** 2021-10-14

**Authors:** Magdalena Kapłan, Kamila Klimek, Ewa Jabłońska-Ryś, Aneta Sławińska, Anna Stój

**Affiliations:** 1 Institute of Horticulture Production, University of Life Science, 28 Głęboka Street, 20-612 Lublin, Poland; magdalena.kaplan@up.lublin.pl; 2 Department of Applied Mathematics and Informatics, University of Life Science, 28 Głęboka Street, 20-612 Lublin, Poland; 3 Department of Plant Food Technology and Gastronomy, University of Life Science, 8 Skromna Street, 20-704 Lublin, Poland; ewa.jablonska-rys@up.lublin.pl (E.J.-R.); aneta.slawinska@up.lublin.pl (A.S.); 4 Department of Biotechnology, Microbiology and Human Nutrition, Faculty of Food Science and Biotechnology, University of Life Sciences in Lublin, 8 Skromna Street, 20-704 Lublin, Poland; anna.stoj@up.lublin.pl

**Keywords:** seedless grape, gibberellic acid (GA_3_), yield, FRAP, DPPH, anthocyanins, correlation

## Abstract

In this study, we determined the effect of hormonization treatment on yield quantity and quality, content of biologically active compounds, and antioxidant activity in fruits and raisins of ‘Einset Seedless’ grapevine. Field studies were conducted in 2017 at Nobilis Vineyard (50°39′ N; 21°34′ E) in the Sandomierz Upland. Analytical studies were carried out in the Laboratory of the University of Life Sciences in Lublin. Hormonized fruits and raisins, which were dried at 40 °C in a food dryer for 7 days, were the experimental material. It was shown that the application of the hormonization treatment had a significant effect on yield size and quality. The hormonization treatment and the form of plant material analyzed had a significant effect on the content of biologically active compounds and the antioxidant activity in ‘Einset Seedless’ grapevine fruits and raisins. The concentration of applied gibberellic acid had a significant effect on the levels of acidity, content of anthocyanins, and antioxidant activity determined with the FRAP and DPPH methods. The application of the multivariate analysis technique showed that, in the fresh fruits and raisins, the level of biologically active compounds and antioxidant activity in the case of the 200 mg∙GA_3_∙L^−1^ concentration and in the control combination was similar but differed significantly in the case of the 300 mg∙GA_3_∙L^−1^ application.

## 1. Introduction

Of all the grapes produced in the world, 80% of the harvest is used to make wine, 13% are dessert grapes, and the rest are used to make raisins [1]. A prerequisite for the profitability of dessert grape production is to adapt the size of its fruits to the high demands of the market, i.e., to obtain the best quality. An important attribute of dessert grapes is also the absence of seeds [2].

Of all the seedless cultivars known in Poland and recommended for outdoor cultivation, ‘Einset Seedless’ is the best, mainly due to its prolificacy, frost resistance, and berry flavor. The fruits of this variety can be used for raisin production and fresh consumption as dessert grapes. However, the natural berry weight of ‘Einset Seedless’ (±2 ÷ 3 g) is not sufficient for commercial use as table grapes [3].

In recent years, new agrotechnical possibilities have emerged to produce very good-quality fruits of these cultivars through exogenous application of gibberellic acid, which stimulates cell division, accelerates flowering, and increases fruit size [4,5,6]. Many studies have shown that the use of gibberellins in parthenocarpic fruit production is very effective [7,8,9,10,11,12]. This is very important, because customers expect grapes that are uniform and reproducible, have equal size and shape and uniform coloration of all berries, and have increased resistance during transport. In addition to improving the yield size and quality of parthenocarpic grapevine varieties, gibberellic acid applications significantly influence berry hardness and elasticity [13]. GA_3_-treated grapes are more resistant to cracking caused by rain, especially at harvest time. Conscious application of gibberellic acid can optimize the yield of seedless grape cultivars by increasing fruit size and improving grape structure. The effectiveness of the treatment itself largely depends on the timing of the treatment, taking into account the developmental stage of the flowers or buds, the concentration of gibberellic acid applied, and the conditions after application [14].

To increase berry and cluster size and compactness, gibberellic acid has been used for many years in countries with long cultivation traditions: in India [15], USA [16,17], Thailand [18], Brazil [19,20], Greece [21], Chile [22], Spain [23], Poland [6,11,12,24], and Jordan [25]. Despite many experiments and studies, there are no clear guidelines on the dose and number of applications of this compound.

Due to its taste and dietetic properties, grapevine fruit is a valuable source of biologically active substances, i.e., vitamins (A, B1, B2, C, PP), minerals (potassium, phosphorus, calcium, iron, boron, magnesium), pectins, dyes, tannins, oils, easily assimilable carbohydrates, amino acids, fruit acids, and fiber. The most important group of health-promoting compounds present in grapes comprises polyphenols: flavonoids, phenolic acids, flavones, flavonols, flavanones, catechins, and anthocyanin pigments [26,27,28,29].

Grape consumption significantly reduces the risk of cardiovascular disease and cancer [30,31,32]. This is due, among other things, to the presence of secondary metabolites such as polyphenols, which have antioxidant, anti-inflammatory, anticancer, antiplatelet, neuroprotective, vasodilator, and immune system-enhancing effects. This is related to the ability of polyphenols to modulate and induce signaling pathways [27,33,34,35,36,37,38]. Furthermore, polyphenols inactivate free radicals and chelate divalent metal ions, thereby reducing their oxidative potential [39].

The qualitative and quantitative composition and distribution as well as the antioxidant activity of polyphenols in grapes are highly variable and depend on the species, cultivar, location in the berries (skin, pulp, seeds, juice), climate and soil conditions in which the bush grows (light exposure, temperature, soil type), agronomic treatments (irrigation, fertilization, use of growth regulators), harvest date, berry maturity, yield and berry size, postharvest conditions, and storage and processing techniques [37,40,41,42,43,44]. Polyphenols are responsible for the main sensory characteristics of plant-derived products and beverages, determining their appearance, especially color, and flavor properties, such as bitterness, astringency, and aroma [45,46].

The aim of this study was to determine the effect of hormonization on yield quantity and quality, the content of biologically active compounds, and antioxidant activity in ‘Einset Seedless’ grapevine fruits and raisins.

## 2. Materials and Methods

Field studies were conducted in 2017 at Nobilis Vineyard (50°39′ N; 21°34′ E) in the Sandomierz Upland. The field experiment was set up in a randomized block design, including 3 combinations with five replications, comprising plots of five grapevines each. Parthenocarpic vines of the ‘Einset Seedless’ cultivar (‘Fredonia’ × ‘Canner’ [47]) were planted in the spring of 2003 at a spacing of 2.0 × 1.0 m (5000 units∙ha^−1^) on a loess-derived podzolic soil containing 2.1% of organic matter. The shrubs were managed with a scaffold consisting of poles and four metal wires stretched at heights of 70, 110, 150, and 190 cm. The plants were managed as a single Guyot twine with a 40 cm high trunk, a single approximately 0.9 m long bed, and a single two-eyed pivot. During the experiment, regular protection against diseases, pests, and weeds was carried out according to the current grapevine protection program. The vines were not irrigated, and the soil pH was 6.2. At the budburst stage, Hydrocomplex fertilizer (12N-11P-18K) was applied by sprinkling at a dose of 300 kg∙ha^−1^, and the other macro- and microelements were supplemented by foliar application as needed. In the field experiment, the effect of gibberellic acid concentration (GA_3_) on the quantity and quality of ‘Einset Seedless’ grapevine fruit yield was evaluated. The solution was prepared using 99% gibberellic acid (Acros OrganicsTM, Thermo Fisher Scientific, Geel, Belgium) and the SILWET Gold (Chemtura Europe Limited, Warsaw, Poland) wetting agent and adhesion promoter at a concentration of 0.015%. The control consisted of plants whose clusters were not treated with gibberellic acid. The solution was prepared just before the treatment. Inflorescences were sprayed with a hand sprayer in the morning, thoroughly covering the stems and flowers. On average, 50 mL of the prepared solution was sufficient to cover all the clusters on one bush very thoroughly. The solutions were applied at concentrations of 200 or 300 mg∙L^−1^. The treatment was performed once during full flowering (70–80% of developed flowers). The level of gibberellic acid concentrations was selected based on the results of our previous research.

In the experiment, the quality of the crop was assessed by analyzing the following parameters: number and weight of bunches, total yield per 1 ha, weight of berries, number of berries per cluster, length of clusters, and length and width of berries. The mean weight and length of clusters were determined by weighing and measuring 15 typical clusters, with 5 clusters randomly taken from each plant. The average weight, number, length, and width of the berries were determined by weighing, counting, and then measuring the berries from five average-sized clusters from each replicate.

The study was conducted in 2017 in the Laboratory of the Department of Plant Food Technology and Gastronomy, Faculty of Food Science and Biotechnology, University of Life Sciences in Lublin. Grapes of the ‘Einset Seedless’ cultivar (‘*Fredonia*’ × ‘*Canner*’ [47], subjected to hormonal treatment with gibberellic acid GA_3_ were the experimental material.

### 2.1. Drying

The samples of the grapes were dried at 40 °C in a food dehydrator (Ezidri Ultra FD 1000, Liberec, Czech Republic) for 7 days.

### 2.2. Total Acidity, Extraction, and Dry Weight Content

Acidity was measured by titration with 0.1 N NaOH according to PN-EN 12147:2000 [48]. The measurements were taken with a class A burette. The level of total titratable acidity was expressed as tartaric acid. Soluble solids were measured using a DR201-95 refractometer (Kruss, Germany). The results were reported as °Bx at 20 °C (PN-A-75101-02:1990/Az1:2002 [49]). The dry weight was determined by drying at 70 °C until constant weight was achieved according to PN-A-75101-03:1990 [50].

### 2.3. Extraction Procedure

The extraction procedure was based on the method described by Kaczmarska et al. [51], with some modifications. One gram of homogenized plant materials (grapes or a 1:1 *w*/*w* mixture of dried raisins and water) was mixed with 5 mL of 80% (*v*/*v*) ethanol. The slurry was shaken for 30 min at room temperature at a speed of 200 rpm. The obtained extracts were centrifuged (MPW 350-R) at 3755× *g* for 15 min. Supernatants were used for the determination of antioxidant activity and total phenolic content.

The extracts for anthocyanin determination were prepared as follows. Mixed grapes or raisins (2 g) were extracted with 10 mL of 80% ethanol acidified with HCl (pH 2) and sonicated for 20 min (Grant, Royston, UK). After centrifugation (4200× *g* for 20 min) (MPW-350R, MPW, Warszawa, Poland), supernatants were concentrated by a rotary evaporator (Laborota 4000 Efficient; Heidolph, Schwabach, Germany) and dissolved in 10 mL of acidified ethanol.

### 2.4. Determination of Total Phenolic Content

The measurement of total phenolics was carried out according to the methodology developed by Singleton and Rossi [52]. The extracts (0.2 mL) were mixed with tenfold diluted Folin and Ciocalteu reagent (0.8 mL). The mixtures were incubated for 3 min at room temperature. Then, 1.25 mL of 7% Na_2_CO_3_ was added, and the mixtures were left to stand for 30 min in the dark at room temperature. The absorbance was read at 725 nm (Helios Gamma; Thermo Fisher Scientific, Waltham, MA, USA) against a blank sample. The phenolic content was expressed as gallic acid equivalent (GAE) per 100 g of fruit fresh mass (FW).

### 2.5. Determination of Ferric Reducing Antioxidant Power (FRAP)

The ferric reducing antioxidant power of the fruit extracts was determined using a FRAP assay following the method proposed by Benzie and Strain [53]. The FRAP reagent was prepared by mixing 300 mM acetate buffer at pH 3.6 with 2,4,6-tri(2-pyridyl)-1,3,5-triazine (TPTZ) (Sigma-Aldrich, St. Louis, MO, USA) solution (10 mM TPTZ in 40 mM HCl) and a 20 mM FeCl_3_∙6H_2_O solution at a 10:1:1 ratio. Assay solutions were prepared by mixing 1.9 mL of the FRAP reagent with 0.1 mL of the fruit extract. After 15 min of incubation (water bath W610; LABOPLAY, Bytom, Poland) at 37 °C in the dark, the absorbance was measured at 593 nm using a Helios Gamma apparatus (Thermo Fisher Scientific, Waltham, MA, USA). The results were reported as µmol of Trolox equivalents (TE) per 1 g of fruit fresh mass (FW).

### 2.6. Determination of DPPH Radical-Scavenging Activity

The DPPH (2,2-diphenyl-1-picrylhydrazyl) radical-scavenging activity was assayed following the method developed by Choi et al. [54]. Each 0.2 mL of the ethanolic extract was mixed with 0.8 mL of a 0.2 mM DPPH ethanolic solution. The mixture was shaken (TK3S; Kartell, Noviglio, Italy) for 15 s and left to stand for 15 min in the dark. The absorbance was measured at 520 nm using a Helios Gamma apparatus (Thermo Fisher Scientific, Waltham, MA, USA). The antioxidant activity was expressed as µmol of Trolox equivalents (TE) per 1 g of fruit fresh mass (FW).

### 2.7. Determination of Total Anthocyanin Content

Total anthocyanin content (TA) was determined using the pH differential method proposed by Wrolstad [55] with some modifications. Then, 250 µL of the extract was diluted with 1 mL of two different buffers: 0.025 M potassium chloride pH 1.0 and 0.4 M sodium acetate pH 4.5. After 15 min of incubation at room temperature, the absorbance (A) was measured at 526 and 700 nm (Helios Gamma; Thermo Fisher Scientific, Waltham, MA, USA). The results were calculated as follows:

A = (A_526_ − A_700_) pH 1.0 − (A_526_ − A_700_) pH 4.5

The content of total anthocyanins (TA) was calculated as follows:

TA = (A × MW × DF × 1000)/(ε × P),

where A is absorbance, DF is the dilution factor, ε is the molar absorptivity coefficient (20,200 cm^−1^ mg^−1^ for malvidin 3-glucoside–M3G) [56], MW is the molecular weight (493.2 for M3G), and P is the cuvette path length (1 cm). The total anthocyanin content was expressed as mg M3G 100 g^−1^ of the fresh weight of grapes or raisins.

All the measurements were taken in triplicate.

The results obtained in this test were statistically analyzed using two-factor analysis of variance and Tukey confidence intervals. Inference was based on a significance level of *p* < 0.05. Correlations between qualitative grape parameters were estimated by counting Pearson correlation coefficients. Multivariate analysis techniques were applied to show similarities within groups such that homogeneous objects were in grouped one cluster. The results of cluster analysis were summarized using a dendrogram. All statistical analyses were performed using SAS Enterprise Guide 5.1 software.

## 3. Results and Discussion

On average, the number of clusters per bush, irrespective of the combination used, was over 19 and did not differ significantly between each other (Table 1).

The gibberellic acid applications had a significant effect on the grapevine yield. The GA_3_ concentration significantly modified the yield, which increased as the GA_3_ concentration increased. Similar relationships were shown by Dimovska et al. [2], who evaluated ‘Flame Seedless’ (*Vitits vinifera* L.) after application of 5, 10, and 20 mg∙GA_3_∙L^−1^.

The hormonal treatment had a significant effect on the grape weight, as the gibberellic acid-treated grapes were significantly heavier than the control. A significant effect of the gibberellic acid concentration on the assessed quality parameter was found, as the clusters treated with 200 mg∙GA_3_∙L^−1^ were significantly heavier than those treated with 300 mg∙GA_3_∙L^−1^. A beneficial effect of gibberellic acid on cluster weight was shown by Habrooks et al., Lu, Casanova et al. and Abu-Zahra [16,17,23,25].

The exogenous applications of gibberellic acid had a beneficial effect on the number of berries per cluster; this effect in the case of application of 300 mg∙GA_3_∙L^−1^ was significant. There was no significant effect of the applied concentration on the trait studied.

The weight of one berry ranged from 1.86 to 2.06 g and differed significantly among the studied combinations (Table 1). The statistical analysis showed a significant effect of the hormonization treatment on the studied trait, while there was no significant effect of the concentration of applied gibberellic acid on the weight of one berry. This is consistent with the results reported by other researchers, e.g., Halbrooks and Mortensen [16], Zabadal and Dittmer [57], and Casanova et al. [23], who showed that gibberellic acid application exerted a significant effect on berry weight. A negative effect of GA_3_ applied at doses of 50 and 300 mg∙GA_3_∙L^−1^ was shown by Lu [17].

The hormonization treatment significantly influenced the size of grapes, which was reflected in the length and width of the cluster (Table 2). The concentration of gibberellic acid had a significant effect on the grape length, whereas no such relationship was shown in the case of the grape width.

The obtained results indicate that the hormonization treatment had a significant effect on the length and width of the ‘Einset Seedless’ grape berries, as the gibberellic acid-treated clusters formed significantly longer and wider berries than the control (Table 2). The concentration of gibberellic acid had a significant effect on the studied quality parameters. Dimovska et al. [2] evaluating the above parameters obtained different results at most of the GA_3_ concentrations applied.

**Figure 1:**
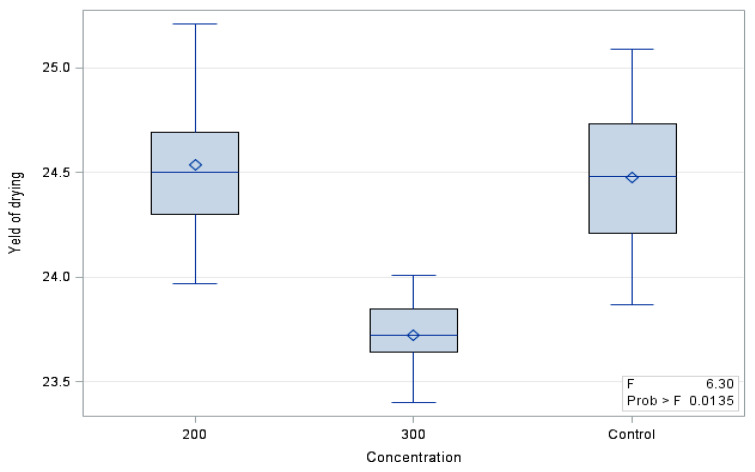
Drying efficiency of ‘Einset Seedless’ fruit.

The drying efficiency of the ‘Einset Seedless’ fruits differed significantly between the evaluated combinations (Figure 1). It was shown that the evaluated parameter in the control grapes (24.48%) and in grapes treated with gibberellic acid at the dose of 200 mg∙GA_3_∙L^−1^ (24.53%) did not differ significantly between each other and was significantly higher than in fruits treated with 300 GA_3_∙L^−1^ (23.72).

**Figure 2:**
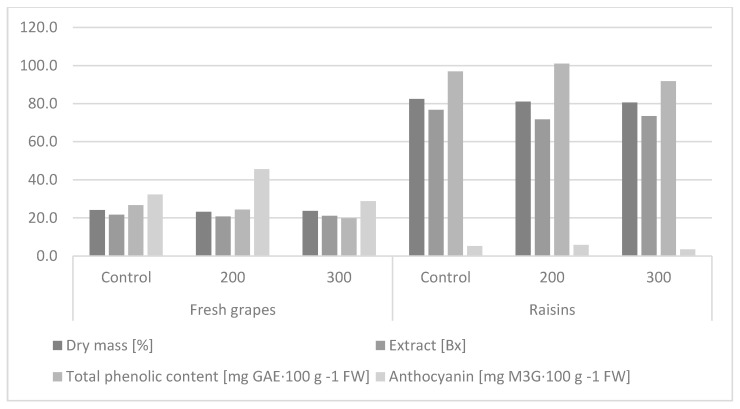
Effect of the hormone treatment on dry matter, extract level, and total phenolic and anthocyanin content in fruits and raisins of the ‘Einset Seedless’ cultivar.

Table 3 presents a two-way analysis of variance. The first example (A) concerns the effect of the GA_3_ concentration level, on the assessed parameters, regardless of the type of raw material, and in case (B), the impact of the type of plant material was considered at the level of the parameters assessed, regardless of the combination used. This combination was considered the mean of the controls, 200 mg∙GA_3_∙L^−1^ and 300 mg∙GA_3_∙L^−1^. The hormonization treatment significantly reduced the dry matter of the ‘Einset Seedless’ grapevine fruits. There was no significant effect of the concentration of gibberellic acid on the studied trait. The fresh fruits had significantly lower dry weight than after drying (Table 3, Figure 2).

**Figure 3:**
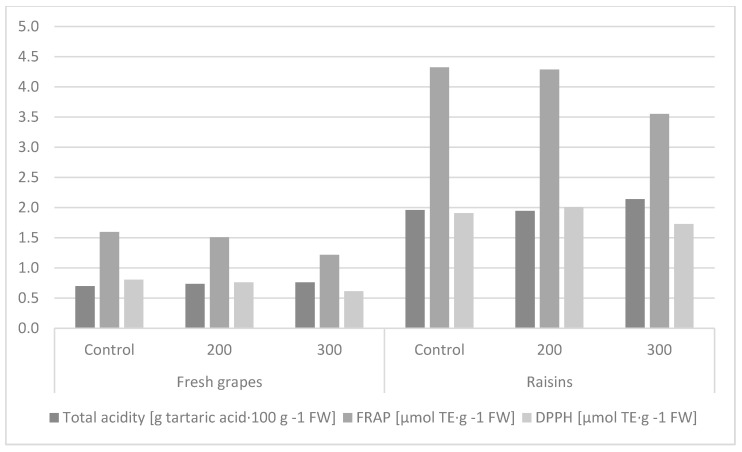
Effect of the hormone treatment on the level of acidity and antioxidant activity in fruits and raisins of the ‘Einset Seedless’ cultivar determined with the FRAP and DPPH methods.

Grape acidity is correlated with flavor due to the presence of tartaric and malic acids, which account for up to 90% of the acids contained in these fruits [58,59]. The total acidity of the hormone-treated fruits differed significantly. It was shown that the control fruits and fruits treated with 200 mg∙GA_3_∙L^−1^ had significantly higher total acidity than the fruits sprayed with gibberellic acid at a concentration of 300 mg∙GA_3_∙L^−1^. It was found that the fresh fruits had significantly lower total acidity than after drying (Table 3, Figure 3). In the available literature, the information on the effect of hormonization treatment on the total acidity level is quite inconsistent. A study conducted by Koka [60] showed no significant effect of GA_3_ application on the acidity of ‘Cardinal’ grapes. An adverse effect of hormonization treatment on the acidity level was shown by Al-Atrushy [61] and Rachna and Singh [62].

Sugars are one of the most important components that determine the quality of fruit, responsible for its sweet taste. The ratio of sugars to organic acids in fruit determines the final taste of grapes [59]. The statistical analysis showed that, regardless of the GA_3_ concentration, the extract level in the hormonized fruits was significantly lower than in the control (Table 3, Figure 2), which confirms previous studies [24]. There was no significant effect of the concentration of gibberellic acid on the studied trait, and similar relationships were shown during observations in 2010–2012 [12]. Dimovska et al. [2] applied GA_3_ at concentrations of 5, 10, and 20 mg∙GA_3_∙L^−1^, and did not show its significant effect on extract content in ‘Flame Seedless’ fruits. In the study conducted by Al-Atrusha [61], the total sugar level increased significantly with an increasing GA_3_ concentration. The present study showed that the level of extract in the fresh fruits was 21.14%, which was significantly lower than that in the raisins (73.96%).

The antioxidant activity of grapevine raw materials is related to the presence of secondary metabolites, such as phenolic acids, anthocyanins, flavonoids, and tannins. The phenolic content in the ‘Einset Seedless’ grapevine fruits was not significantly affected by the hormonization treatment and the concentration of GA_3_ applied. It was shown that the raisins obtained from the fruits of the studied grapevine cultivar were characterized by more than four times significantly higher level of phenolic compounds than the fresh fruits (Table 3, Figure 2).

The antioxidant activity of the fruit extracts determined with the FRAP and DPPH methods significantly depended on the concentration of GA_3_. Fruits treated with 200 mg∙GA_3_∙L^−1^ were characterized by significantly higher antioxidant activity than those treated with 300 mg∙GA_3_∙L^−1^. The level of antioxidant activity expressed by the DPPH parameter in the control combination did not differ significantly from the others, while similar relationships were shown between the control and application of 200 mg∙GA_3_∙L^−1^ in the case of the FRAP parameter. The fresh fruits were characterized by significantly lower antioxidant activity than the raisins (Table 3, Figure 3). Gougoulias and Masheva [63] showed a positive effect of the hormonization treatment on the increase (from 16 to 42%) in the level of antioxidant activity of seedless grape varieties.

The hormone treatment significantly increased the anthocyanin content only when 200 mg∙GA_3_∙L^−1^ was applied (Table 3, Figure 2). The study conducted by Dimovska et al. [2] showed no significant effect of the hormonization treatment on the level of these compounds in fruits of the ‘Flame Seedless’ cultivar. Fresh fruits had more than seven times significantly higher levels of anthocyanins than raisins.

The article presents a Pearson’s correlation analysis in order to determine the influence or lack thereof between the parameters if the chemical composition of biologically active compounds of ‘Einset Seedless’ grape. Pearson’s coefficient for biologically active compounds in the ‘Einset Seedless’ grapevine fruits indicates a strong significant correlation between the DPPH parameter and FRAP, and the total phenolic content and the DPPH and FRAP parameters. A strong significant negative correlation was observed between dry weight and anthocyanin content, total acidity and phenolic content, and DPPH and FRAP parameters (Table 4).

The analysis of Pearson’s coefficient for parameters determining biologically active compounds in the ‘Einset Seedless’ grapevine raisin showed a strong significant correlation between DPPH and FRAP, the total phenolic level and the DPPH and FRAP parameters, and the anthocyanin content and FRAP. A strong negative correlation was observed between total acidity and FRAP. A strong correlation relationship between the considered features within the clusters was expected. Higher extract content in fruit and products made from them is closely related to a higher dry matter value. In turn, the antioxidant properties, determined by FRAP and DPPH methods, largely depend on the overall content of phenolic compounds, including anthocyanins. Such dependencies have been demonstrated in many research studies, including by Kaczmarska et al. [50] (Table 5).

**Figure 4:**
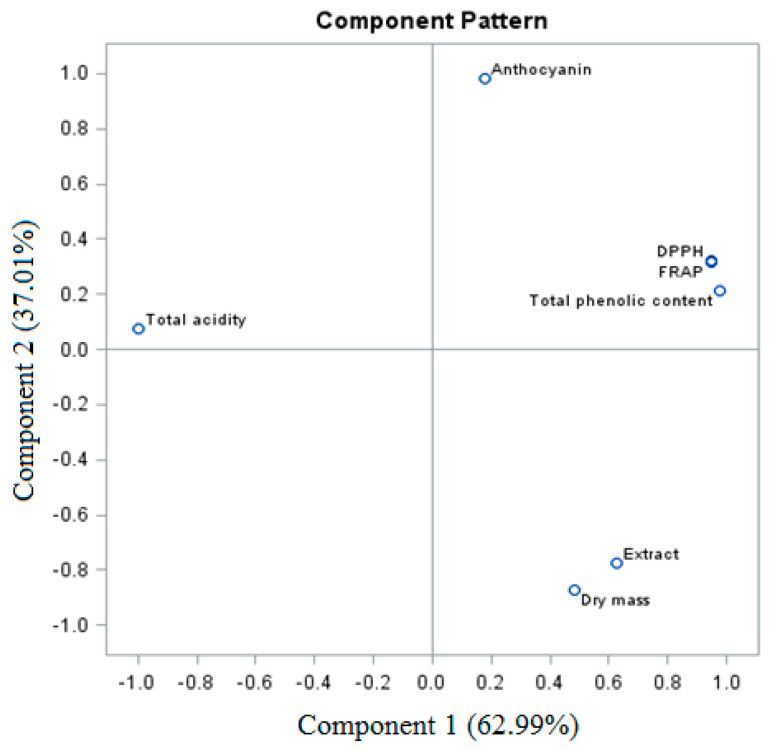
PCA analysis of fresh ‘Einset Seedless’ fruits against biologically active compounds.

The sum of PC (PC1 and PC2) of the total trait variable for the fresh ‘Einset Seedless’ fruits was 100% (for PC1, 62.99%, and for PC2, 37.01%, respectively) (Figure 4). Considering the analysis of biologically active compounds and antioxidant activity of the fresh fruits, a similarity was observed between certain parameters, which formed four groups. The first group is the relationship between dry weight and extract, while the second group is the relationship between the total phenolic content and the DPPH and FRAP parameters. Groups three and four are anthocyanins and total acidity.

**Figure 5:**
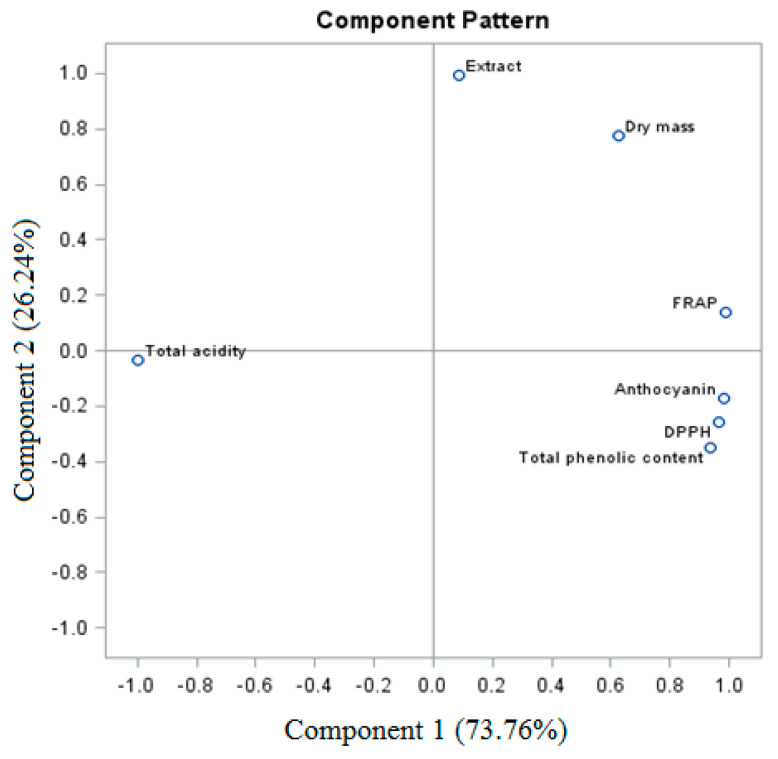
PCA analysis of ‘Einset Seedless’ raisins against biologically active compounds.

The sum of PC of the total variable for the analyzed grape raisins ‘Einset Seedless’ was 100% (for PC1 73.76% and for PC2 26.24%, respectively) (Figure 5). The analysis of the biologically active compounds and antioxidant activity of raisins revealed a similarity between certain parameters, which formed three groups. The first one is the relationship between total phenolic content, anthocyanins, and the DPPH and FRAP parameters. The second relationship was shown between dry weight and extract. The third group was the total acidity.

**Figure 6:**
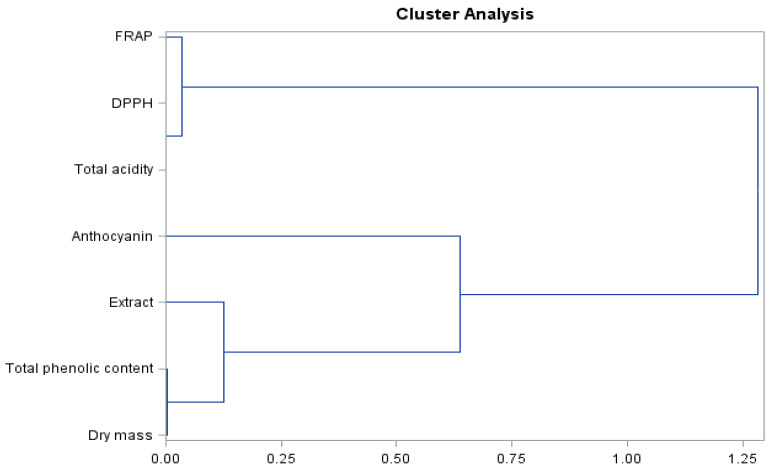
Branching-tree diagram for biologically active compounds and antioxidant activity of ‘Einset Seedless’ fruits irrespective of the concentration of gibberellic acid.

As can be seen from the presented dendrogram (Figure 6) for biologically active compounds and antioxidant activity of the fruits of the studied grapevine cultivar, there are three clusters and an object determining the level of total acidity, which is not included in the dendrogram. A high similarity is shown by group 1—dry matter, total phenolic level, and extract; group 2 are anthocyanins; and group 3 are FRAP and DPPH parameters.

**Figure 7:**
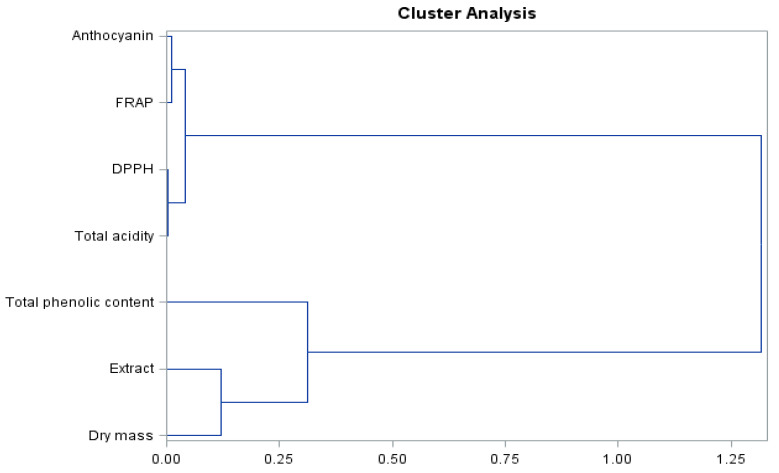
Branching-tree diagram for biologically active compounds and antioxidant activity of ‘Einset Seedless’ raisins regardless of the concentration of gibberellic acid.

The presented dendrogram (Figure 7) allowed us to determine the similarity of the level of biologically active compounds and antioxidant activity of the ‘Einset Seedless’ raisins. Based on the results, two pairs of clusters showing clear similarities were identified. The analysis revealed that anthocyanins together with the FRAP parameter showed a clear similarity, as did the acidity level and the DPPH parameter. These compounds formed a common object, indicating a high similarity. Similar relationships were shown for the extract and dry matter contents, which formed an object combining with the total phenolic content. The formed objects merge into one at the distant level.

**Figure 8:**
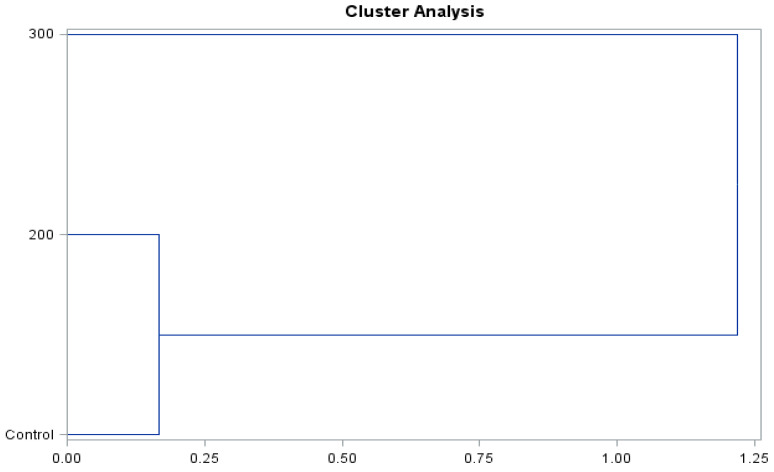
Branching-tree diagram for the hormonal treatment and the concentration of gibberellic acid applied in ‘Einset Seedless’ fruits.

**Figure 9:**
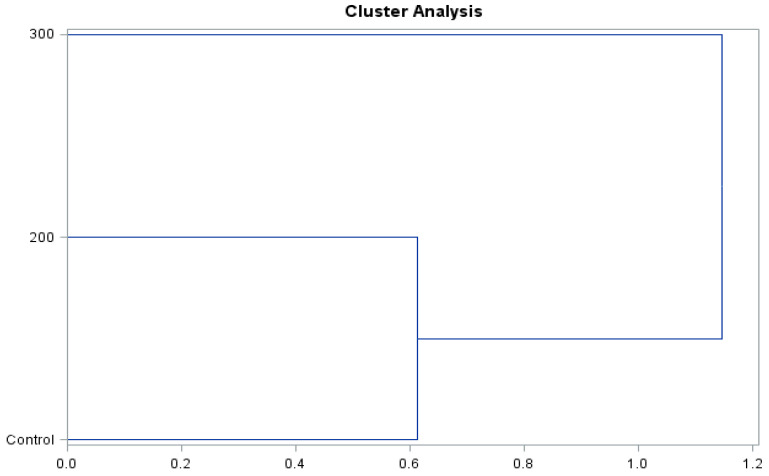
Branching-tree diagram for the hormonal treatment and the concentration of gibberellic acid applied in ‘Einset Seedless’ raisins.

The presented dendrograms (Figure 8 and Figure 9) allowed us to determine the similarity of the effect of the concentrations of gibberellic acid on the content of biologically active compounds and antioxidant activity of the ‘Einset Seedless’ fruits and raisins. Irrespective of the type of the examined raw material, it was shown that the level of biologically active compounds and antioxidant activity in the case of the concentration of 200 mg∙GA_3_∙L^−1^ and the control combination was similar but differed substantially in the case of the application of 300 mg∙GA_3_∙L^−1^. The above-mentioned relationships occurred in the case of both fresh fruits and raisins.

## 4. Conclusions

The application of the hormonization treatment in the ‘Einset Seedless’ grapevine cultivar had a significant effect on yield quantity and quality. Grapes treated with gibberellic acid were characterized by a significantly higher yield, as well as weight, length, and width of grapes, than that of the control. There was a significant effect of the concentration of gibberellic acid applied on the yield, weight, and length of grapes as well as the length and width of berries. Grapes treated with 300 mg∙GA_3_∙L^−1^ were characterized by significantly better parameters of the yield size and quality than those exposed to 200 mg∙GA_3_∙L^−1^.

The hormonization treatment and the form of the plant material analyzed had a significant effect on the content of biologically active compounds and antioxidant activity of the ‘Einset Seedless’ fruits and raisins. The hormonization treatment significantly reduced the level of dry matter and extract cleary no significant differences were found between 200 mg∙GA_3_∙L^−1^ and 300 mg∙GA_3_∙L^−1^. The concentration of gibberellic acid had a significant effect on the levels of acidity, anthocyanins, and antioxidant activity determined with the FRAP and DPPH methods. The increase in GA_3_ concentration from 200 mg∙L^−1^ to 300 mg∙L^−1^ had a negative impact on the size of the parameters tested.

The fresh fruits were characterized by significantly lower levels of dry matter, acidity, extract, phenols, and antioxidant activity determined with the FRAP and DPPH methods than the raisins. A reverse relationship was shown in the case of the anthocyanin levels.

The application of the multivariate analysis technique showed that, in both the fresh fruits and the raisins, the level of biologically active compounds and antioxidant activity in the case of the 200 mg∙GA_3_∙L^−1^ concentration and the control combination was similar, but it was significantly different in the case of the 300 mg∙GA_3_∙L^−1^ application.

## Figures and Tables

**Table 1:** Effect of gibberellic acid on the size yield of ‘Einset Seedless’ grapevine.

Combination	Cluster Vine^−1^	Yield kg∙Vine^−1^	Cluster		Berry Weight, g
			Weight, g	Berries Cluster^−1^	
Control	19.30 ± 0.25 ^A^*	2.34 ± 0.22 ^C^	121.2 ± 12.24 ^C^	65.2 ± 6.04 ^B^	1.86 ± 0.11 ^B^
200 mg∙GA_3_∙L^−1^	19.28 ± 0.16 ^A^	2.58 ± 0.13 ^B^	133.6 ± 7.58 ^B^	67.5 ± 2.51 ^AB^	1.98 ± 0.08 ^A^
300 mg∙GA_3_∙L^−1^	19.26 ± 0.15 ^A^	2.76 ± 0.16 ^A^	143 ± 5.73 ^A^	69.4 ± 2.79 ^A^	2.06 ± 0.08 ^A^
*p*-value	0.5203	<0.0001	<0.0001	0.0048	<0.0001

* Mean values marked with the same letters do not differ significantly at *p* > 0.05; ns—not significant. A B C is an indication and the difference between averages.

**Table 2:** Effect of gibberellic acid on the quality of yield of ‘Einset Seedless’ grapevine cultivar.

Combination	Cluster		Berry	
	Length, cm	Width, cm	Length, mm	Width, mm
Control	15.32 ± 0.08 ^C^*	9.10 ± 0.29 ^B^	17.88 ± 0.22 ^C^	15.28 ± 0.43 ^C^
200 mg∙GA_3_∙L^−1^	15.92 ± 0.78 ^B^	9.76 ± 1.15 ^A^	18.74 ± 0.18 ^B^	15.6 ± 0.46 ^B^
300 mg∙GA_3_∙L^−1^	16.68 ± 1.01 ^A^	10.58 ± 1.62 ^A^	19.58 ± 0.82 ^A^	15.98 ± 0.37 ^A^
*p*-value	<0.0001	<0.0001	<0.0001	<0.0001

* Mean values marked with the same letters do not differ significantly at *p* > 0.05; ns—not significant. A B C is an indication and the difference between averages.

**Table 3:** Effect of the hormone treatment on the content of biologically active compounds and antioxidant activity in ‘Einset Seedless’ fruits and raisins.

		Dry Mass *	Total Acidity	Extract	Total Phenolic Content	FRAP	DPPH	Anthocyanin
Concentration (A)	Control	53.32 ± 31.93 ^A^**	1.30 ± 0.68 ^B^	49.19 ± 30.16 ^A^	61.79 ± 38.71 ^A^	2.95 ± 1.49 ^A^	1.35 ± 0.61 ^AB^	18.69 ± 14.91 ^B^
	200	52.16 ± 31.71 ^B^	1.34 ± 0.66 ^B^	46.22 ± 27.97 ^B^	62.67 ± 42.16 ^A^	2.89 ± 1.53 ^A^	1.38 ± 0.68 ^A^	25.69 ± 21.86 ^A^
	300	52.17 ± 31.16 ^B^	1.45 ± 0.75 ^A^	47.25 ± 28.69 ^B^	55.83 ± 40.06 ^A^	2.38 ± 1.31 ^B^	1.17 ± 0.63 ^B^	16.09 ± 13.83 ^B^
*p*-value		<0.0001	<0.0001	<0.0001	0.1349	<0.0001	<0.0001	<0.0001
Type of plant material (B)	Grapevine fruits	23.71 ± 0.42 ^B^	0.73 ± 0.03 ^B^	21.14 ± 0.74 ^B^	23.61 ± 3.15 ^B^	1.43 ± 0.18 ^B^	0.72 ± 0.09 ^B^	35.49 ± 8.03 ^A^
	Raisins	81.41 ± 0.88 ^A^	2.01 ± 0.11 ^A^	73.96 ± 2.42 ^A^	96.58 ± 8.19 ^A^	4.05 ± 0.44 ^A^	1.88 ± 0.21 ^A^	4.85 ± 1.12 ^B^
*p*-value		<0.0001	0.0048	0.0006	<0.0001	0.0008	0.0328	<0.0001
(A∙B)		<0.0001	0.0614	0.0114	0.6499	0.171	0.6024	<0.0001

* Dry mass %. Total acidity (g tartaric acid∙100 g^−1^ FW). Extract (°Bx). Total phenolic content (mg∙GAE∙100 g^−1^ FW) FRAP (µmol TE∙g^−1^ FW) DPPH (µmol TE∙g^−1^ FW) Anthocyanin (mg M3G∙100 g^−1^ FW) ** Mean values marked with the same letters do not differ significantly at *p* > 0.05; ns—not significant. A B is an indication and the difference between averages.

**Table 4:** Correlation coefficient for biologically active compounds in the ‘Einset Seedless’ fruits and their significance.

	Dry Mass	Total Acidity	Extract	Total Phenolic Content	FRAP	DPPH	Anthocyanin
Dry mass	1	−0.4317	0.4952	0.2897	0.1743	0.1580	−0.7140
		0.2460	0.1753	0.4496	0.6537	0.6847	0.0307
Total acidity	−0.4317	1	−0.3123	−0.7513	−0.6011	−0.5993	−0.0511
	0.2460		0.4132	0.0196	0.0869	0.0881	0.8962
Extract	0.4952	−0.3123	1	0.0453	0.0360	0.1147	−0.5596
	0.1753	0.4132		0.9078	0.9267	0.7690	0.1172
Total phenolic content	0.2897	−0.7513	0.0453	1	0.8967	0.9054	0.4008
	0.4496	0.0196	0.9078		0.0011	0.0008	0.2851
FRAP	0.1743	−0.6011	0.0360	0.8967	1	0.9833	0.4244
	0.6537	0.0869	0.9267	0.0011		<0.0001	0.2549
DPPH	0.1580	−0.5993	0.1147	0.9054	0.9833	1	0.4053
	0.6847	0.0881	0.7690	0.0008	<0.0001		0.2792
Anthocyanin	−0.7140	−0.0511	−0.5596	0.4008	0.4244	0.4053	1
	0.0307	0.8962	0.1172	0.2851	0.2549	0.2792	

**Table 5:** Correlation coefficient for biologically active compounds in the ‘Einset Seedless’ raisins and their significance.

	Dry Mass	Total Acidity	Extract	Total Phenolic Content	FRAP	DPPH	Anthocyanin
Dry mass	1	−0.4883	0.7712	0.0200	0.4528	0.1225	0.4119
		0.1823	0.0150	0.9593	0.2210	0.7535	0.2707
Total acidity	−0.4883	1	0.0035	−0.2716	−0.6458	−0.3074	−0.7914
	0.1823		0.9928	0.4795	0.0063	0.4211	0.0111
Extract	0.7712	0.0035	1	−0.0130	0.1596	−0.0429	−0.1010
	0.0150	0.9928		0.9736	0.6818	0.9127	0.7960
Total phenolic content	0.0200	−0.2716	−0.0130	1	0.7952	0.9286	0.2885
	0.9593	0.4795	0.9736		0.0104	0.0003	0.4515
FRAP	0.4528	−0.6458	0.1596	0.7952	1	0.8669	0.6691
	0.2210	0.0063	0.6818	0.0104		0.0025	0.0487
DPPH	0.1225	−0.3074	−0.0429	0.9286	0.8669	1	0.4209
	0.7535	0.4211	0.9127	0.0003	0.0025		0.2593
Anthocyanin	0.4119	−0.7914	−0.0011	0.2885	0.6691	0.4209	1
	0.2707	0.0111	0.7960	0.4515	0.0487	0.2593	

## Data Availability

Not applicable.
